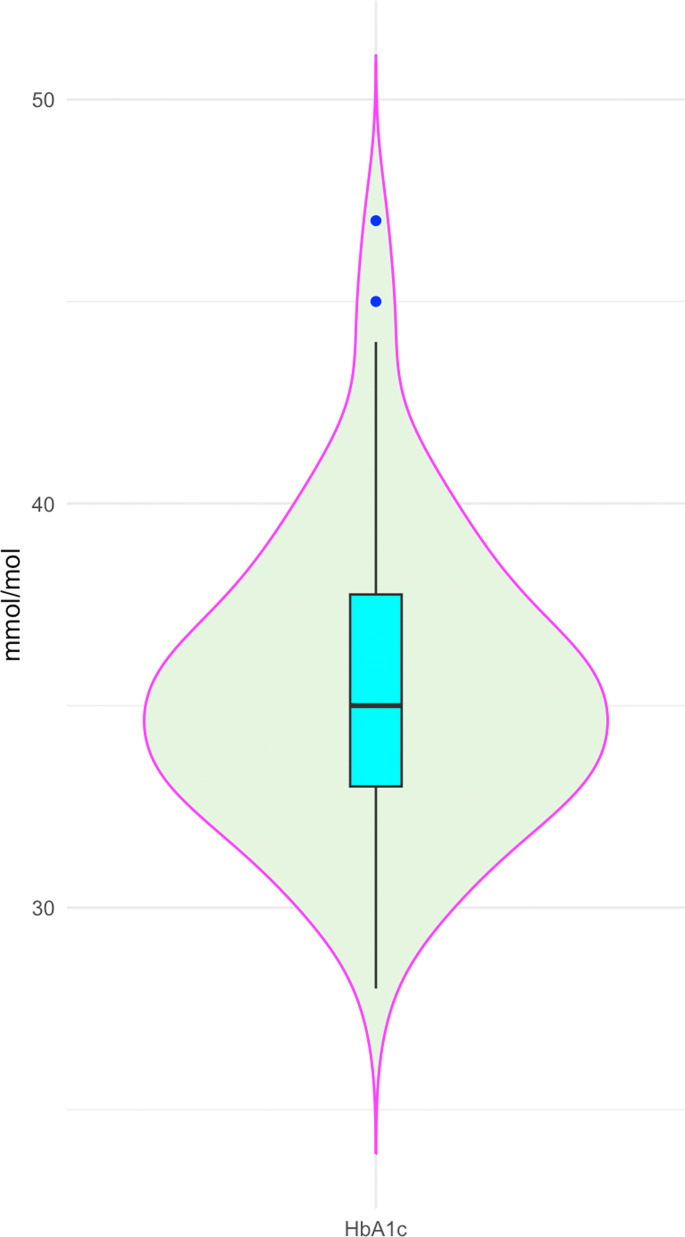# Low carbohydrate high fat-diet in real life; A descriptive analysis of cardiovascular risk factors

**DOI:** 10.1016/j.ijcrp.2025.200384

**Published:** 2025-03-08

**Authors:** Henrik Hagström, Linda Nyström Hagfors, Rikard Hedelin, Mattias Brunström, Krister Lindmark

**Affiliations:** a Department of Public Health and Clinical Medicine, Umeå University, Umeå, Sweden; b Department of Food, Nutrition and Culinary Science, Umeå University, Umeå, Sweden; c Department of Clinical Sciences, Danderyd Hospital, Karolinska Institute, Stockholm, Sweden

**Keywords:** "Diet, carbohydrate-Restricted", "Diet, ketogenic", "Diet, high-fat", "Heart disease risk factors"

## Abstract

**Aims:**

Low Carbohydrate High Fat (LCHF) diets are popular for weight loss or glucose control. The main source of energy in such diets is fat but the composition of nutrients varies This study aims to investigate dietary variations in a real-world LCHF population and its associations with cardiovascular risk factors.

**Methods:**

We recruited 100 volunteers who considered themselves adherent to a LCHF diet. Their last 14 days of dietary intake was assessed using diet history interviews. Validation of energy intake against expenditure was made using activity monitors. Predictive variables for the linear regression models were selected using stepwise bidirectional assessment of Akaike information criterion (AIC).

**Results:**

Energy intake (E%) from carbohydrates was low, 8.7 E%, and fat was the main replacement. Dietary cholesterol was associated with higher total cholesterol, low-density lipoprotein (LDL), and high-density lipoprotein (HDL). Dietary sodium intake was associated with higher blood pressure. Protein intake was associated with lower diastolic blood pressure but also with lower HDL. The intake of dietary fibre was associated with lower LDL and total cholesterol but with higher hemoglobin A1c (HbA1c). The intake of carbohydrates and saturated fatty acids (SFA) was not associated with any of the outcome variables.

**Conclusion:**

In this LCHF population, variations in intake of carbohydrates and saturated fatty acids could not predict any aspects of the cardiovascular risk profile. Lower fibre intake and higher cholesterol and sodium intake predicted a less favorable cardiovascular risk profile.

**What is already known on this topic**

Low carbohydrate diets have shown weight loss properties short term but concerned have also been raised regarding negative effects on cardiovascular risk factors. How the nutritional variation within the diet impacts cardiovascular risk factors is not fully known.

**What this study adds**

This study found no association between carbohydrate levels or saturated fatty acid levels and the risk factors analyzed. Cholesterol and salt intake was associated with worse lipid profile and blood pressure and fibre intake was associated with slightly better lipid profile.

**How this study might affect research, practice, or policy**

This observational study supports the recommendations in nutritional guidelines regarding fibre, cholesterol, and salt intake even in the setting of a low carbohydrate diet.

**Lay summary**

This study examined how different ways of eating a Low Carbohydrate High Fat (LCHF) diet impact factors related to heart health. The LCHF diet, popular for weight loss and blood sugar control, involves eating high amounts of fats and very low amounts of carbohydrates.

We studied 100 people who followed a LCHF diet, analyzing their food intake over the past two weeks. We aimed to identify which parts of the diet were associated with risk factors of heart disease.

The findings revealed that eating more cholesterol was linked to higher levels of both good (HDL) and bad (LDL) cholesterol in the blood. Higher salt intake was associated with increased blood pressure and higher levels of triglycerides. Increased protein intake was associated with lower diastolic blood pressure but also to lower levels of good cholesterol. Lower fibre intake was linked to higher levels of bad cholesterol (LDL) and total cholesterol, though it was also linked to lower blood sugar levels. The amount of carbohydrates and saturated fats consumed did not seem to affect any risk factors in this study.

In summary, for individuals on a LCHF diet, eating more fibre while reducing cholesterol and salt intake might improve the cardiovascular risk factor profile while the amount of carbohydrates and saturated fats did not show a significant impact in this study.

## 1 Introduction

LCHF diets have been proposed for weight loss or blood glucose control in diabetes, but many other personal reasons and beliefs may influence the choice of diet. Dietary recommendations specifically tailored for the patient preferring a LCHF diet does not exist.

The main feature of these diets is reduction in dietary carbohydrates. Carbohydrate levels are often as low as <10 % of energy intake (E%) and potentially ketogenic [1,2]. Studies have shown that a LCHF diet can be used to facilitate weight loss and glucose control [[3], [4], [5], [6]]. The limitation is often a short follow up period of 6–12 months. This is important as the effect of dietary interventions often seems to diminish over time [7,8].

Carbohydrates are replaced predominantly with fats. The LCHF diet is commonly encouraged to include foods that are considered natural. This means that unprocessed foods are promoted and foods rich in saturated fats are often preferred over low-fat alternatives. Saturated fat and cholesterol intake have been linked to higher risk for cardiovascular disease whereas unsaturated fats have shown benefits [[10], [11], [12], [13]]. A few small randomized controlled trials have shown a marked increase in LDL cholesterol among healthy subjects eating a LCHF diet compared to a normal reference diet [14,15]. Weight loss is associated with lowering of LDL-levels which may balance out some of the change in LDL when the diet results in weight loss [16].

We have studied a group of volunteers eating a self-reported LCHF-diet. In a previous publication, we have described our study population in detail regarding their nutritional intake [9]. We found that a low intake of carbohydrates and high intake of fat is a reality among motivated individuals. The median intake of saturated fats in our study population was over 30 E%. That is more than three-fold the recommended maximum intake in Sweden [17]. The negative effects of saturated fats are downplayed in the LCHF-community but a well composed LCHF diet with predominantly unsaturated fats is possible to achieve. One of our hypotheses is that a higher intake of saturated fatty acids (SFA) is associated with higher LDL in a real-life LCHF-population.

Dietary fibre intake in low-carbohydrate diets (LCDs) is often lower than recommended [18]. In our study it was about half of what is recommended in the Nordic Nutritional Recommendations [9,17]. Low intake of fibre has been linked to increased risk of cardiovascular disease and diabetes [19,20].

Salt content in the diet and its effects on cardiovascular risk is a matter of concern. The mean intake of salt in the general population is above what is stated in the nutritional recommendations [21]. The effect of salt reduction on blood pressure seems linear and appears largest among those with existing hypertension [22]. This has led to a recent lowering in the recommended level of daily intake [23]. The intake of salt in our study population was lower than in the general population but higher than recommended [9].

It is of interest to investigate various aspects of the LCHF diet in relation to cardiovascular risk factors, as this could contribute to more tailored dietary advice. The aim of this study was to explore variations in nutritional composition and their association with cardiovascular risk factors within a LCHF-population.

## 2 Methods

### 2.1 Study design, setting, and participants

One hundred volunteers who considered themselves adherent to a LCHF-diet since at least 3 months were recruited and a cross-sectional study was done. The participants were free from lipid lowering medication and known familiar hyperlipidemia. The study population and methods for dietary assessment as well as validation have been previously described in detail [9]. This study was conducted in accordance with the Helsinki declaration, approval from the local Ethics Committee was obtained (approval number 2017/23–31), and all participants signed a written informed consent form.

### 2.2 Patient involvement

Patients were not directly involved in designing this study. The study questions were however conceived in the meetings with patients. The study was made possible by the volunteers that generously contributed with their time.

### 2.3 Variables and measurements

The participantś weight, length, waist, and hip circumference were measured. Blood pressure was measured three times in a seated position using a manual aneroid sphygmomanometer. Blood and urine samples were taken. Routine blood test was analyzed the same day and blood samples were stored in a biobank for later analysis. All measurements and samples for all study subjects were performed by the same research nurse. Total Energy expenditure (TEE) was estimated by activity monitoring for 7 days using SenseWear Armband Pro3 (SWA) (BodyMedia Inc., Pittsburgh, PA.) The nutritional composition of the diet was assessed through diet history interviews covering the previous two weeks. All interviews were conducted and nutritionally calculated by the same dietitian.

The energy intake (EI) was validated through comparison with TEE. Implausible levels of EI were assessed using the Goldberg cut off updated by Black [24]. Participants with plausible levels of EI were considered acceptable reporters. Participants reported if they were currently weight stable or not. The results are based on the acceptable reporters, while in the sensitivity analyses, both all participants and those who reported weight stability were used.

Basal metabolic rate (BMR) was calculated using the Mifflin St Jeor method [25]. This method has shown a slightly better accuracy compared to other methods [26]. Food Intake Level (FIL) was calculated by dividing EI by BMR and Physical Activity Level (PAL) was calculated by dividing TEE by BMR.

### 2.4 Statistical methods

Statistical analyses were conducted in R, (v4.3.2; R Core Team 2023) using libraries mice (v3.16.0; van Buuren 2023), and car (v3.1-2; Weisberg S 2019).

Shapiro-Wilks's test was used to assess normal distributions. Variables were considered non-normally distributed if the p-value was below 0.05. Normally distributed data are presented as mean + - SD. Non-normally distributed data are presented as median (25–75 percentiles).

One missing data point each in HbA1c, LP(a) and TEE, was imputed using Multivariate Imputation by chained equation (MICE). Data was assumed missing at random. All the variables analyzed in this study was used to form a predictor matrix which was used by the algorithm to impute the missing data through Predictive Mean Matching (PMM) in five iterations. Since there was only three missing datapoints, no sensitivity analyses were made.

Stepwise linear regression modelling was performed using the step() function in R.

Lipid profile, systolic and diastolic blood pressure, hemoglobin A1c (HbA1c) were used as outcome variables. Age, sex, Body Mass Index (BMI), PAL, FIL, TEE, EI, fibre intake, cholesterol intake, sodium intake as well as proportion of energy (E%) from carbohydrates, protein, fat, Monounsaturated Fatty Acids (MUFA), Polyunsaturated Fatty Acids (PUFA), (SFA), and alcohol were considered as explanatory variables.

After testing for multicollinearity using Variance Inflation Factors (VIF), PAL, FIL, total fat intake, MUFA and PUFA were removed. A possible explanation for the multicollinearity is that including all macronutrient components as well as subgroups of fats results in too much overlapping information. FIL includes reported energy intake, weight and age which is also information that may be overlapping. None of the remaining predictors had a VIF of more than 6 and was therefore considered acceptable for the analysis in the aspect of multicollinearity [27].

The step model was done bi-directionally, starting forward from a clean intercept-only model. Each predictive variable was then added sequentially. Akaike information criterion (AIC) was used to find the next best fitting predictor [28]. AIC rewards a model with good fit but penalizes many predictive variables to prevent overfitting. The step function iterates and adds a predictive variable if it improves the AIC value. When there is no more improvement to be gained the model removes any predictive variable if it improves the AIC. When there is no more improvement of the AIC to be gained the model is selected as the best.

## 3 Results

The characteristics of our study population are described in Table 1 and Fig. 1, Fig. 2. The proportion of women was about two thirds. About half of the participants had a BMI of 25 kg/m^2^ or more. Median systolic blood pressure was 120 mmHg and 14 of the participants had a systolic blood pressure of 140 mmHg or higher. Median Hemoglobin A1c (HbA1c) was 35.0 (33.0–38.0) mmol/mol and only one of the participants had a HbA1c of 48 mmol/mol or higher. Median total cholesterol and LDL was 6.2 (5.5–7.0) and 3.8 (3.0–4.5) mmol/L respectively. Median HDL was 1.8 (1.6–2.3) mmol/L. Distribution of lipid components, HbA1c and blood pressure is shown in detail in the violin plots in Fig. 1, Fig. 2.

**Table 1:** Study population.

Study population characteristics		
	Acceptable reporters (n = 83)	All (n = 100)
Age – years	48.9 (39.7–59.6)	48.7 (40.2–59.9)
Women - %	55	62
Smokers - %	0	0
Body Mass Index – kg/m^2^	25.1 (23.0–28.5)	25.7 (23.1–28.5)
Waist Hip Ratio	0.9 (0.8–0.9)	0.9 (0.8–0.9)

Systolic Blood Pressure – mmHg	120.3 (112.2–136.0)	121.0 (112.9–132.9)
Diastolic Blood Pressure– mmHg	80.7 (76.2–87.3)	81.8 (76.3–88.1)

HbA1c - mmol/mol	35.0 (33.0–38.0)	36.0 (33.0–38.0)

Total cholesterol - mmol/L	6.2 (5.5–7.0)	6.3 (5.8–7.1)
LDL - mmol/L	3.8 (3.0–4.5)	4.0 (3.0–4.6)
HDL - mmol/L	1.8 (1.6–2.3)	1.8 (1.6–2.3)
Triglycerides - mmol/L	0.8 (0.6–1.0)	0.8 (0.6–1.0)
Lp(a) - nmol/L	14.0 (0.0–59.5)	14.5 (0.0–42.5)

Data presented as median (25–75 percentiles).

**Fig. 1:**
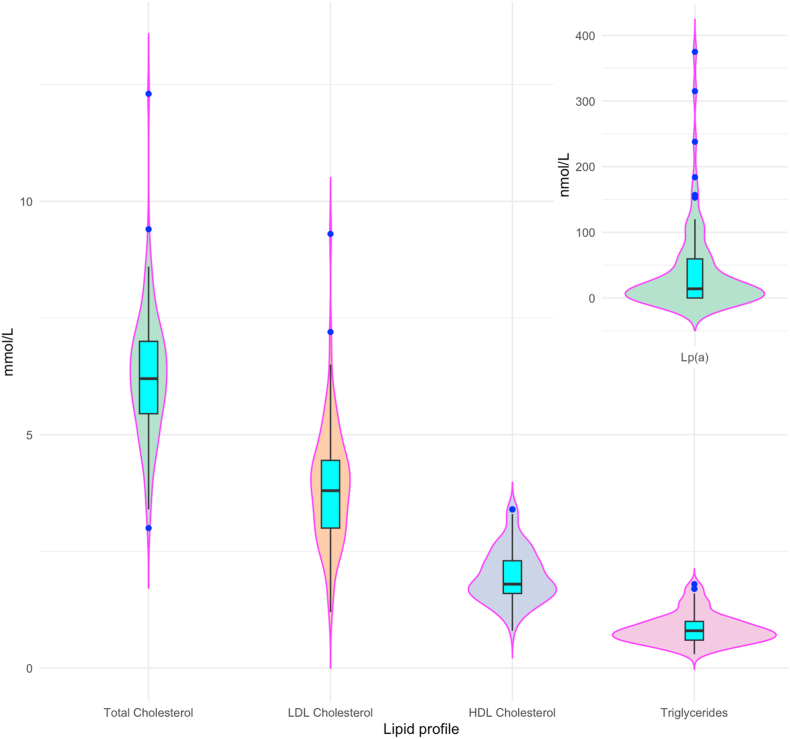
Distribution of lipids (acceptable reporters). Legend:Distribution of lipids.Box plot shows median and 25–75 percentiles.Violin plots show distribution densityLDL – Low Density Lipoprotein, HDL – High Density Lipoprotein, Lp(a) – Lipoprotein (a).

**Fig. 2:**
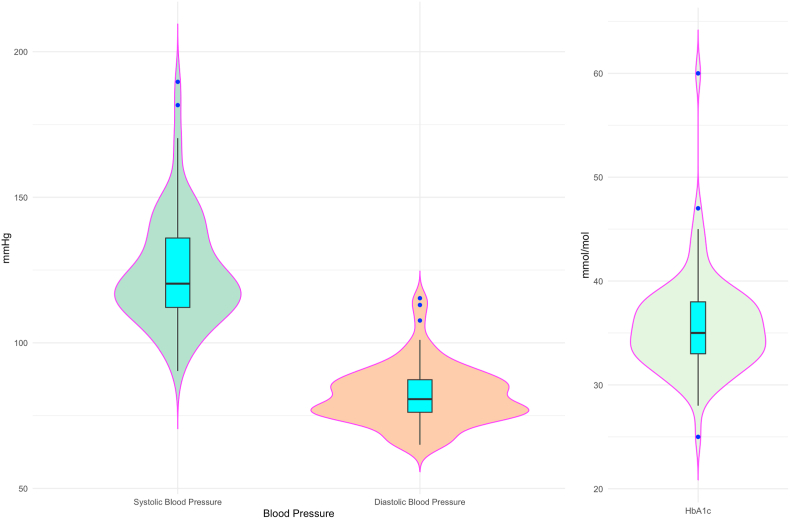
Blood pressure and HbA1c (acceptable reporters). Legend:Distribution of systolic and diastolic blood pressure.Box plot shows median and 25–75 percentiles.Violin plots show distribution densityHbA1c – Hemoglobin A1c.

The median intake of carbohydrates was low, 8.7 E% (Table 2). This was compensated mainly with a high level of energy intake from fats, 72.3 E%. Dietary fibre intake was also low. The absolute intake was in median 13 g/day and relative to energy intake it was on average 1.6 ± 0.7 g/MJ. Only three of the acceptable reporters reached the recommended intake level of 3 g/MJ and none of them had an intake level of carbohydrates below 10 E%.

**Table 2:** Summary of dietary components (acceptable reporters).

Summary of dietary components (acceptable reporters)				
	g/day		E%	
	(median)	(25–75 percentiles)	(median)	(25–75 percentiles)
Carbohydrates	44.6	(33.4–53.9)	8.7	(6.4–11.0)
Added sugar	1.8	(0.5–3.3)	0.5	(0.1–0.7)
Fibre	13.0	(8.7–17.3)		
Fat	161.5	(127.0–194.9)	72.3	(67.8–76.0)
SFA	72.7	(55.0–94.5)	32.0	(28.1–37.9)
MUFA	54.9	(41.7–66.2)	23.7	(22.0–26.2)
PUFA	19.1	(14.2–25.0)	8.4	(6.7–10.1)
Cholesterol	0.7	(0.5–0.8)		
Protein	83.3	(68.0–103.5)	17.0	(15.0–19.2)
Sodium	2.8	(2.4–3.6)		
Alcohol	3.7	(0.0–9.9)	1.3	(0.0–3.5)

SFA - Saturated Fatty Acids, MUFA - Mono Unsaturated Fatty Acids, PUFA - Poly Unsaturated Fatty Acids.

Higher age was associated with higher LDL, HDL, Total cholesterol, HbA1c and blood pressure (Table 3). Male sex was associated with lower HDL, higher triglycerides and higher HbA1c. Higher BMI was associated with lower HDL and total cholesterol as well as higher triglycerides and diastolic blood pressure.

**Table 3:** Summary of linear regression models (acceptable reporters).

Summary of linear regression models (acceptable reporters)								
Betacoefficients (positive or negative direction of effect)								
	LDL	HDL	TC	TGC	LP(a)	HbA1c	SBP	DBP
	(mmol/L)	(mmol/L)	(mmol/L)	(mmol/L)	(nmol/L)	(mmol/mol)	(mmHg)	(mmHg)
Age (years)	**0.02**	**0.01**	**0.03**			**0.16**	**0.65**	**0.24**
Male sex		**−0.52**		**0.24**		**2.68**		
BMI (kg/m^2^)		**−0.04**	**−0.06**	**0.02**				**0.54**
EI (100 kcal/day)				**−0.02**				
TEE (100 kcal/day)								
Carbohydrate (10 E%)								
Protein (10 E%)		**−0.40**						**−7.18**
Fibre (g/day)	**−0.05**		**−0.05**			**0.17**		
SFA (10 E%)								
Cholesterol (g/day)	**1.35**	**0.50**	**1.9**					
Salt (g/day)				**0.03**			**1.41**	**1.66**
Alcohol (10 E%)				**0.26**		**−5.28**		
Adjusted R^2^	**0.26**	**0.40**	**0.24**	**0.30**	**ns**	**0.28**	**0.37**	**0.26**

LDL - Low Density Lipoprotein, HDL - High Density Lipoprotein, TC - Total Cholesterol, TGC - Triglycerides, LP(a) - Lipoprotein (a), HbA1c - Hemoglobin A1c, SBP - Systolic Blood Pressure, DBP - Diastolic Blood Pressure, EI - Energy Intake, TEE - Total Energy Expenditure, SFA - Saturated Fatty Acids, BMI - Body Mass Index.

Dietary cholesterol was associated with higher total cholesterol, LDL and HDL. Dietary sodium intake was associated with higher blood pressure and higher triglycerides. Protein intake was associated with lower diastolic blood pressure but also with lower HDL. Fibre intake was associated with lower LDL and total cholesterol but with higher HbA1c. Alcohol intake was associated with lower HbA1c but higher triglycerides.

Carbohydrate and SFA intake showed no association with any of the outcome variables. Energy intake and energy expenditure was not associated with any meaningful change in any of the outcomes. Energy intake did show statistical significance in the model predicting triglycerides, but the beta coefficient was close to zero.

The results above are based on analyses of acceptable reporters (n = 83). Sensitivity analyses of all participants and participants reporting weight stability were made and are available in the supplementary material. When analyzing all participants, male sex was associated with higher systolic blood pressure and protein intake was associated with lower systolic blood pressure (Table S3). These associations were not found when considering only acceptable reporters or participants reporting weight stability (Table 3 and S4).

## 4 Discussion

As a group, our participants were strictly adherent to a LCHF-diet as discussed in a previously published article [9]. The results are based on the acceptable reporters. However, sensitivity analyses with all participants and those reporting weight stability did not differ significantly.

Median blood pressure and HbA1c was normal. The median total cholesterol level was higher than in northern Sweden [29]. LDL was in the higher range of the normal interval. On the other hand, the HDL levels were also on the higher end of the normal range and triglycerides were low. Overall, the risk profile in the study group was quite favorable, albeit with higher than optimal total cholesterol and LDL.

BMI, sex, and age was often found to contribute to the models. The effect of higher BMI, male sex and higher age was in general towards worse lipid profile and higher blood pressure, which is in-line with epidemiological studies in general populations [30,31]. The exceptions were that higher age was associated with higher HDL-cholesterol and higher BMI was associated with lower total cholesterol. In general populations, BMI has been associated with higher total cholesterol [31]. The positive relationship between age and HDL has been found previously in a cross-sectional study but was questioned in a longitudinal arm [32].

The energy intake from fats was very high in our population [9]. About twice as much as the recommended intake level in the Nordic countries [17]. SFA-rich foods are often encouraged in the LCHF-community. Our main hypothesis was that a LCHF-diet high in SFA would be associated with higher levels of LDL compared to a LCHF-diet with lower intake of SFA. SFA has previously been associated to cardiovascular morbidity but did not provide any predictive power in any of our models [10]. These findings are not in line with current literature and conclusions are hard to draw [10]. The intake of SFA was very high in our population and none of the participants had an intake level below 10E%. The intake was roughly 2.5 times higher than in the general Swedish population [33]. Despite the high intake level and reasonably large variations, SFA intake still did not predict any of the outcomes.

Dietary cholesterol was found to contribute to the predictive power of the models for LDL, HDL, and total cholesterol. In all cases, higher dietary intake of cholesterol was associated with higher levels of the outcome variable. This is in agreement with meta-regression analyses of interventional trials [11]. The importance of a recommended upper limit of dietary cholesterol have been debated. The available observational evidence of dietary cholesterol and egg consumption on cardiovascular outcomes is heterogenous and does not point in a single direction, and the cholesterol consumption in the general population have been reasonably modest [11]. The intake of cholesterol was however quite high in our population [9,33]. The median intake was 0.7g (0.5–0.9 mg). The most common sources of cholesterol are meat, eggs, and dairy products. The intake level of eggs is not analyzed in our study, but intake of eggs is encouraged in the LCHF community. Cholesterol intake and the effect on mortality has been evaluated in several prospective trials when examining intake of eggs [34,35]. Very few prospective studies have evaluated such high consumption, but the trends point towards a higher risk for all-cause mortality as well as cancer mortality, even though the effect seems less clear in European populations [36].

Interestingly, dietary carbohydrate intake, was not found to contribute in any of the predicting models. Carbohydrate intake is a focal point in a low-carbohydrate diet, and the low levels in the study population may not provide enough variation to detect any correlations with the outcomes.

Higher dietary fibre intake was associated with lower LDL and total cholesterol but also higher HbA1c. The latter finding is somewhat surprising considering that current literature points more towards and anti-diabetic effect of dietary fibre intake [19]. Previous findings do however show the strongest association in diabetics, who are not represented in our study. Several potential mechanisms are in play on the glycemic regulation when dietary fibre and carbohydrates are ingested together [37]. The effects seen are however towards lower post-prandial blood sugar and lower risk of diabetes and cannot explain our finding. The effects of dietary fibre intake in the setting of a low carbohydrate diet are less explored. The European Food Safety Authority, EFSA, suggests that an intake of 4g of dietary fibre per 30g of available carbohydrates is adequate to enjoy the benefits on glycemic control [38]. In our study, median intake of fibre was 8.7g per 30g of carbohydrates, although low, in absolute terms. In our study, HbA1c increased with 0.17 mmol/mol per 1g/day increment of fibre intake, and although statistically significant, there is still a risk that the finding is a play of chance. Intake of fibre has an inverse association with cardiovascular risk in general [20]. Low intake of fibre has been a concern in the LCHF-setting since many of the food items that are rich in fat and low in carbohydrates are also low in fibre. The fibre intake in our study population was about half of the minimum recommended level in Sweden, which may be a problem in people who adhere long-term to a LCHF diet. This data may support a recommendation to increase the intake of greens and nuts rich in fibre to lower the risk of cardiovascular disease, especially in a LCHF-diet.

Higher dietary protein was associated with lower HDL but also lower diastolic blood pressure. The effect of replacing carbohydrates with protein has been examined in a systematic review [39]. The findings showed a reduction in blood pressure which is in line with our findings. The association with lower HDL is contradictory to other studies [40]. In a general dietary pattern, higher protein intake is often associated with a lower carbohydrate intake, which has been shown association with higher LDL [41]. The role of protein intake on HDL in a low carbohydrate setting is less known. The source of protein is also important, where plant-based proteins have been found to be associated with lower cardiovascular risk [42]. Detailed analyses of food groups have not yet been done in our study, but preliminary estimates suggest a large portion of the protein are from an animal origin.

Higher intake of sodium was associated with higher blood pressure. This finding is well in-line with current knowledge of the relationship between sodium intake and blood pressure [43]. The Nordic Nutrition Recommendations 2023 recommend limiting sodium intake to 2.3 g/day, equivalent to about 5.75 g of salt (18). The European Food Safety Authority (EFSA) and European Society of Hypertension (ESH) recommends an even lower intake level of sodium of less than 2g per day based on evidence of cardiovascular risk benefits [44]. This corresponds to 5g of salt per day. The median intake in our study population was about 2.8g of sodium per day. The sodium intake of the Swedish population in general, is well above the recommended intake. In the Swedish Cardiopulmonary bioImage Study (SCAPIS), the mean sodium intake was 2.9g for women and 3.6g for men. One explanation for the lower reported intake in our study population may be that processed foods and bread are discouraged in the LCHF community and these food items are often high in sodium content [21]. However, in the SCAPIS study, the intake of sodium was estimated using sodium excretion in the urine which is a more precise estimation than used in our study [45].

Alcohol intake was associated with higher triglycerides but also lower HbA1c. These relationships have been found in previous studies [46,47].

The aim of this study was to explore associations of nutritional and cardiovascular risk factors. In general, our descriptive models provided R^2^-values of about 25–40 %, which shows that 60–75 % of the variations in the risk factors measured, could not be predicted by the models. Because there are many factors contributing to cardiovascular risk other than dietary factors, this is expected. An umbrella review found that the pooled risk ratios for cardiovascular outcomes and all-cause mortality ranged from about 0.55 to 0.90 depending on the intervention and the outcome analyzed [48]. That may serve as an indicator of the maximum effect one can expect from a dietary intervention. When measuring effect on surrogate markers such as risk factors for cardiovascular disease, one must also consider that the attributable effect of the five most common modifiable risk factors on cardiovascular disease is not close to 100 percent but rather 50–60 % [49].

## 5 Strengths and limitations

The models in our analysis with the best predictive power were selected using the AIC to prevent overfitting and we chose to discuss the results mainly on the acceptable reporters for higher accuracy, although there are benefits to present results in all participants. This explorative data analysis was done with data from a cross-sectional study. Generalization of conclusions should be made with caution, and the study does not allow for any conclusions of causality. It does however provide some insights into the variations of the diet in a LCHF-population and how they correlate with the variations in selected risk factors.

The recruitment of study participants was made using newspaper ads and spread of word. The inclusion criteria included adherence to a LCHF-diet defined by the participant. This way of recruiting carries a risk of selection bias, but it was chosen to best represent a real-life population. The sample size is reasonably large and trying to capture a representative subpopulation eating LCHF in a larger screening study of the general population is hard. Crude comparison of age, sex, BMI and WHR shows an acceptable agreement with the general population besides an overrepresentation of women in our population [50]. The study population was largely normotensive, with comparable levels of blood pressure to the general population in Västerbotten County, Sweden [51]. None of the participants reported smoking which may be indicative for a bias towards a greater interest in health in our study population compared to the general population.

We used a diet history interview with two weeks recall as our method, which provided detailed data on dietary intake. Assessment of nutritional intake is challenging, and memory-based methods have several limitations and have been criticized [52]. Possible sources of error include recall bias, food portion estimation, and the desire to report a diet that may be perceived as correct. To strengthen the methodology, we used the same trained dietitian for all interviews. The diet history interviews conducted in this study were also very thorough, and a strength of this method is that it is based on the specific eating habits of the participants. For example, this study made extensive use of the participants' own recipes for nutritional calculations. Furthermore, the reported dietary intake was validated using activity sensors and showed reasonable agreement between reported energy intake and energy expenditure at a group level [9]. The dietary assessment is a strength of our study even though risk of bias persists.

The results were based on the 83 participants that were classified as acceptable reporters. Sensitivity analyses did not show any major change in the results of the regression analyses apart from a correlation between male sex and systolic blood pressure that remains unexplained.

## 6 Conclusions

In this real-life LCHF population, carbohydrate intake was low, and the small variations were not associated with any of the risk factors studied. The intake of SFA was high but variations herein were not associated with any of the risk factors.

Cholesterol intake, which was high, was associated with worse lipid profile and sodium intake with higher blood pressure, aligning with previous research.

Concerns regarding low fibre intakes in LCHF diets were substantiated by our findings, suggesting that this is a reality at least in this group of LCHF eaters. Lower fibre intake was also associated with worse lipid profile and lower fibre intake have been associated with higher risk of cardiovascular disease in other study populations.

Due to the cross-sectional design of this study, the results should be interpreted as hypothesis-generating rather than conclusive. To establish causality, longitudinal studies are warranted to further explore these associations with cardiovascular disease risk factors.

For individuals following LCHF diets, this study reinforces the guideline recommendations to intentionally include fiber-rich foods in their diet while avoiding excessive salt and cholesterol intake.

## CRediT authorship contribution statement

**Henrik Hagström:** Writing – original draft, Methodology, Formal analysis, Conceptualization. **Linda Nyström Hagfors:** Conceptualization, Supervision, Writing – review & editing. **Rikard Hedelin:** Supervision, Writing – review & editing. **Mattias Brunström:** Methodology, Supervision, Writing – review & editing. **Krister Lindmark:** Conceptualization, Supervision, Writing – review & editing

## Data availability statement

Data from the study will be available upon reasonable request.

## Ethics approval statement

This study was conducted in accordance with the Helsinki declaration. Approval from the Swedish Ethical Review Authority in Umeå was obtained (approval number 2017/23–31), and all participants signed a written informed consent form.

## Funding

The study was financed by grants from the Swedish state under the agreement between the Swedish government and the county councils, the ALF-agreement.

## Declaration of competing interest

The authors declare the following financial interests/personal relationships which may be considered as potential competing interests: Mattias Brustrom reports a relationship with Amarin Pharma Inc that includes: consulting or advisory and speaking and lecture fees. Mattias Brunstrom reports a relationship with AstraZeneca AB that includes: consulting or advisory. Mattias Brunstrom reports a relationship with Medtronic Inc that includes: speaking and lecture fees. If there are other authors, they declare that they have no known competing financial interests or personal relationships that could have appeared to influence the work reported in this paper.

## Figures and Tables

**figs1:**
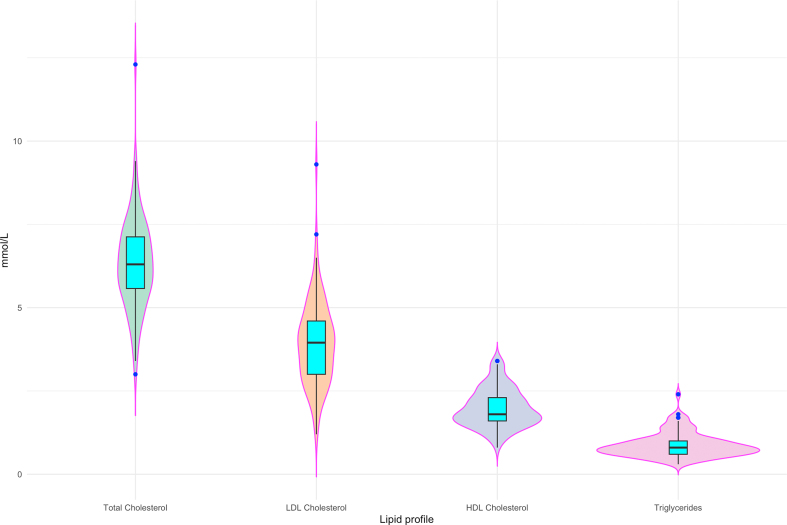


**figs2:**
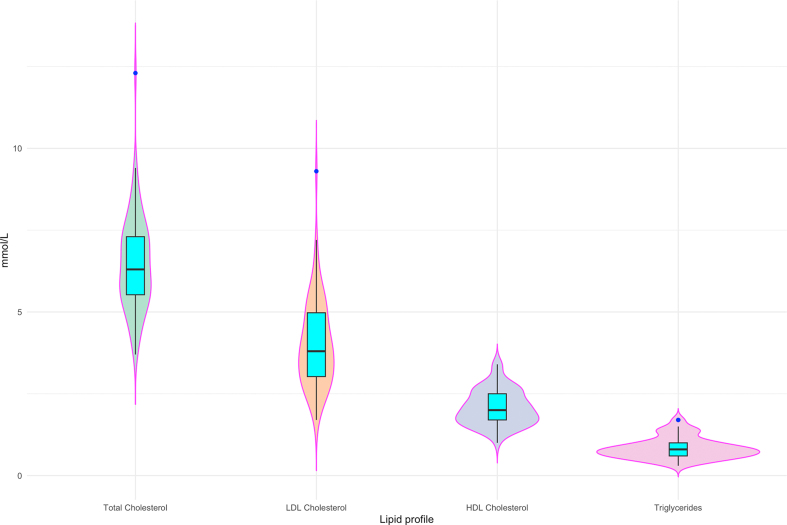


**figs3:**
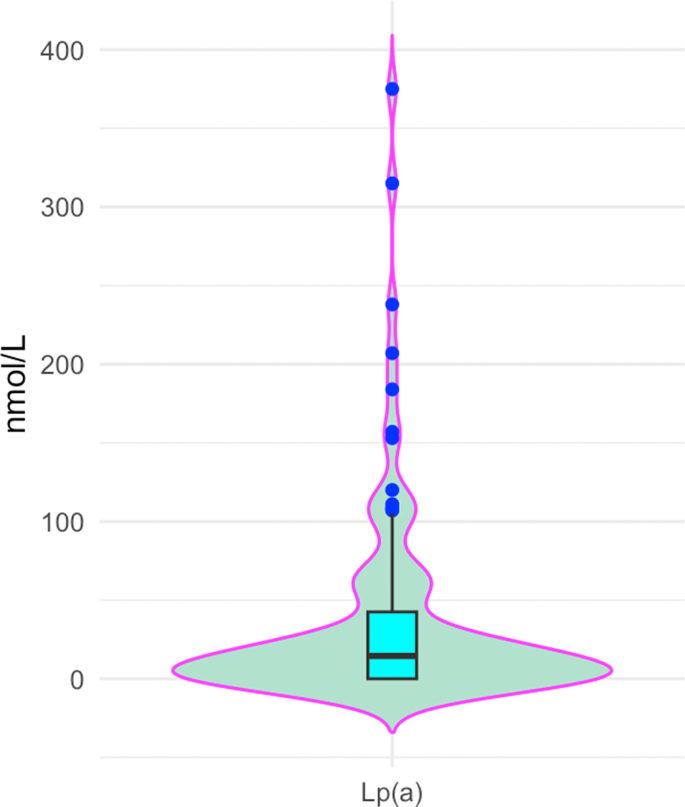


**figs4:**
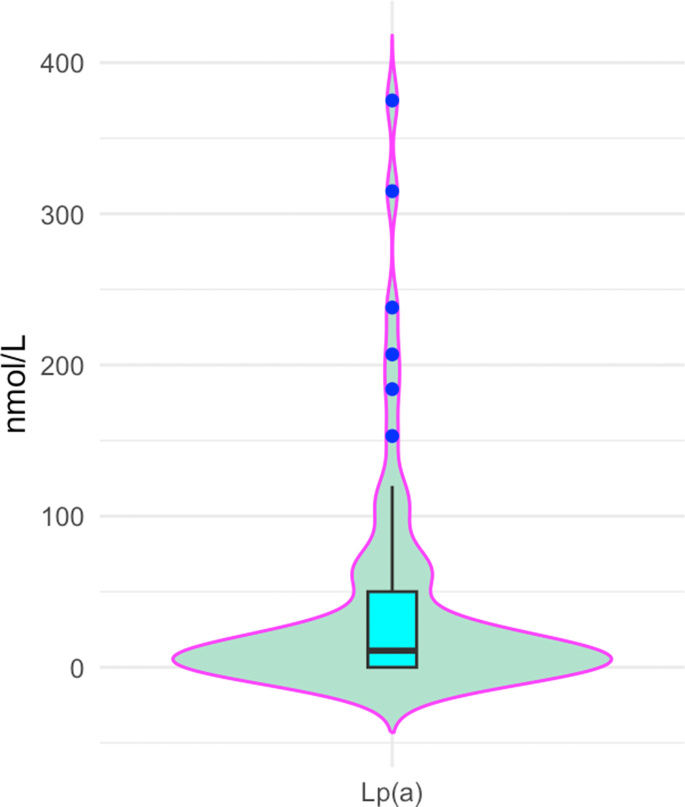


**figs5:**
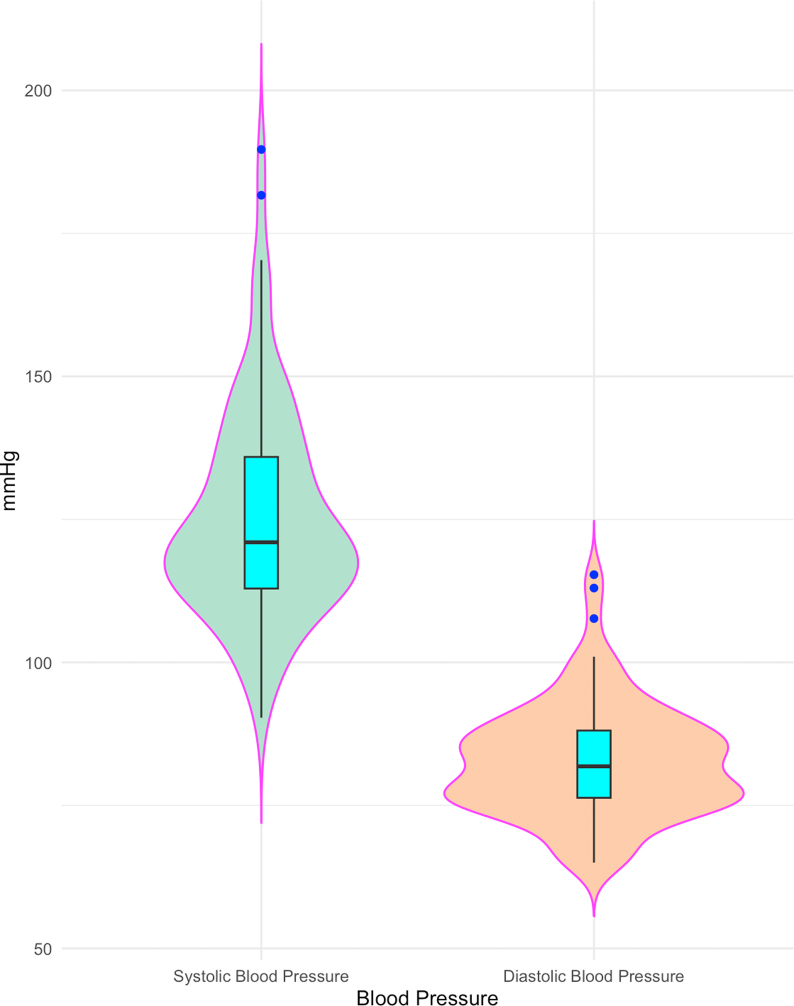


**figs6:**
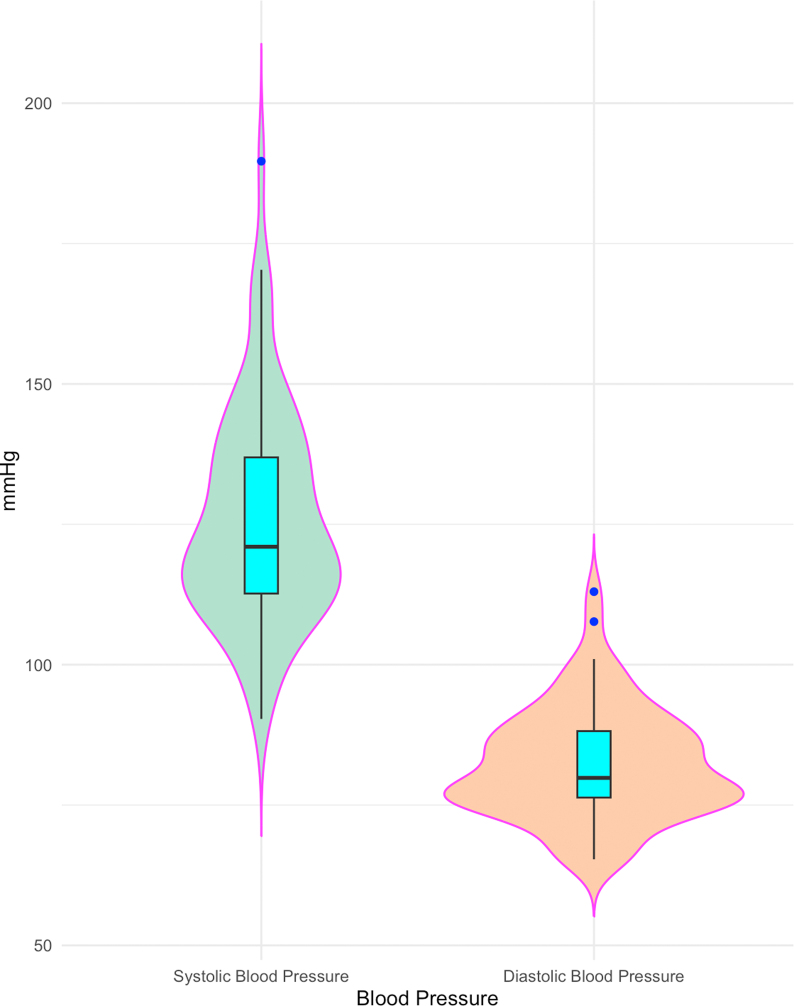


**figs7:**
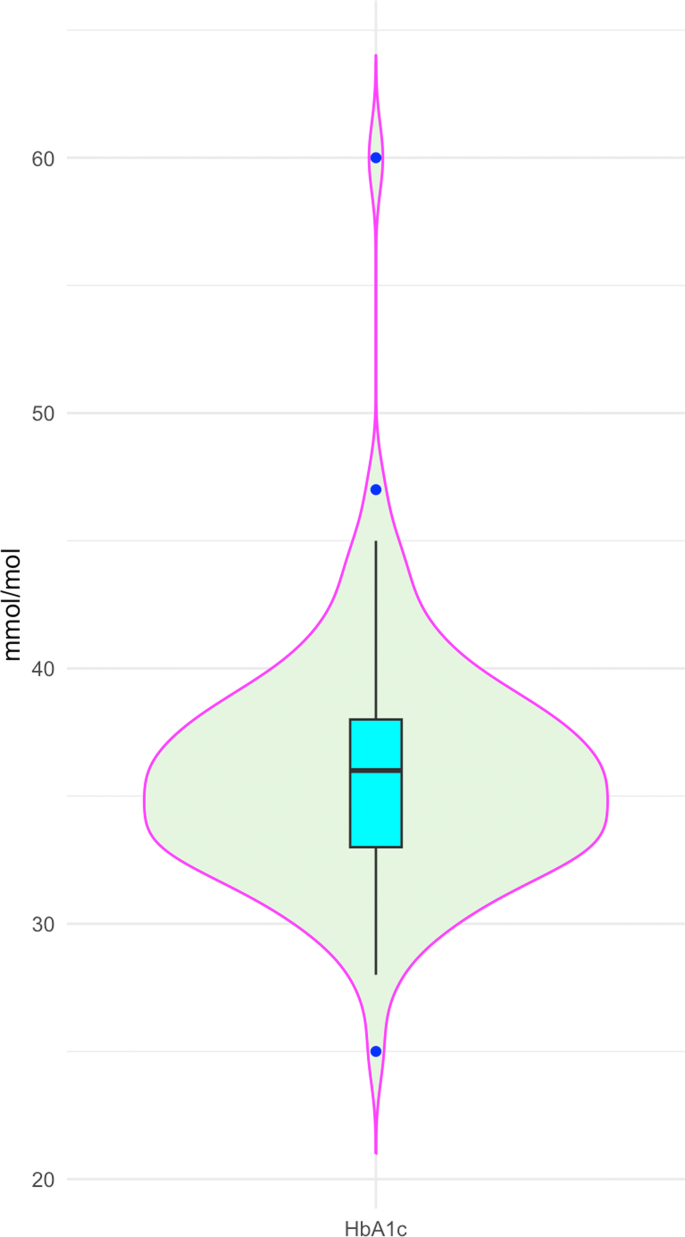


**figs8:**