# Supplementary material for: Low carbohydrate high fat-diet in real life; A descriptive analysis of cardiovascular risk factors
Source: Int J Cardiol Cardiovasc Risk Prev. 2025 Mar 8;25:200384. doi: 10.1016/j.ijcrp.2025.200384 (PMC11957601; doi:10.1016/j.ijcrp.2025.200384)
Supplement: Multimedia component 1 [file mmc1.docx]

# **Supplementary material**

Table S1. Summary of dietary components (all)

| Summary of dietary components (all) | | | |  |
| --- | --- | --- | --- | --- |
|  | g/day |  | E% |  |
|  | (median) | (25-75 percentiles) | (median) | (25-75 percentiles) |
| Carbohydrates | 42.9 | (33.0-54.0) | 8.7 | (6.5-11.1) |
| Added sugar | 1.8 | (0.5-3.6) | 0.3 | (0.1-0.8) |
| Fibre | 12.9 | (8.3-17.5) |  |  |
| Fat | 156.1 | (125.2-194.0) | 72.0 | (67.6-76.2) |
| SFA | 70.7 | (53.3-94.0) | 31.9 | (28.1-38.3) |
| MUFA | 52.5 | (40.1-65.0) | 23.6 | (22.0-25.8) |
| PUFA | 18.7 | (13.7-24.5) | 8.1 | (6.7-9.9) |
| Cholesterol | 0.7 | (0.5-0.9) |  |  |
| Protein | 82.0 | (65.8-102.3) | 16.9 | (14.5-19.1) |
| Sodium | 2.8 | (2.4-3.7) |  |  |
| Alcohol | 4.3 | (0.0-10.3) | 1.4 | (0.0-3.6) |
| *SFA - Saturated Fatty Acids, MUFA - Mono Unsaturated Fatty Acids,* | | | | |
| *PUFA - Poly Unsaturated Fatty Acids* | | |  |  |

Table S2. Summary of dietary components (weight stable)

| Summary of dietary components (weight stable) | | | | |
| --- | --- | --- | --- | --- |
|  | g/day |  | E% |  |
|  | (median) | (25-75 percentiles) | (median) | (25-75 percentiles) |
| Carbohydrates | 43.0 | (34.7-52.9) | 7.6 | (6.0-10.7) |
| Added sugar | 2.0 | (0.5-3.8) | 0.3 | (0.1-0.8) |
| Fibre | 13.0 | (9.2-17.6) |  |  |
| Fat | 158.6 | (130.6-202.4) | 72.3 | (67.9-76.4) |
| SFA | 72.2 | (55.1-96.3) | 31.9 | (27.8-38.9) |
| MUFA | 54.5 | (44.2-69.1) | 24.2 | (22.2-27.1) |
| PUFA | 19.1 | (14.0-24.3) | 7.9 | (6.8-9.6) |
| Cholesterol | 0.7 | (0.5-1.0) |  |  |
| Protein | 85.3 | (68.9-105.6) | 16.5 | (13.9-18.8) |
| Sodium | 2.9 | (2.4-3.8) |  |  |
| Alcohol | 6.7 | (0.3-12.6) | 1.5 | (0.1-4.0) |
| *SFA - Saturated Fatty Acids, MUFA - Mono Unsaturated Fatty Acids,* | | | | |
| *PUFA - Poly Unsaturated Fatty Acids* | | |  |  |

Table S3.

| Summary of linear regression models (all) | | | | |  |  |  |  |
| --- | --- | --- | --- | --- | --- | --- | --- | --- |
| Betacoefficients (positive or negative direction of effect) | | | | | |  |  |  |
|  | LDL | HDL | TC | TGC | LP(a) | HbA1c | SBP | DBP |
|  | (mmol/L) | (mmol/L) | (mmol/L) | (mmol/L) | (nmol(L) | (mmol/mol) | (mmHg) | (mmHg) |
| Age (years) |  | **0.01** | **0.03** |  |  | **0.18** | **0.66** | **0.24** |
| Male sex | **0.58** | **-0.65** |  | **0.31** |  |  | **10.65** |  |
| BMI (kg/m^2^) |  | **-0.06** | **-0.06** | **0.04** |  |  |  | **0.55** |
| EI (100 kcal/day) |  |  |  |  |  |  | **-0.74** |  |
| TEE (100 kcal/day) |  | **-0.03** |  | **-0.02** |  |  |  |  |
| Carbohydrate (10 E%) | |  |  |  |  |  |  |  |
| Protein (10 E%) |  | **-0.34** |  |  |  |  | **-9.90** | **-6.37** |
| Fibre (g/day) | **-0.05** |  | **-0.04** |  |  | **0.12** |  |  |
| SFA (10 E%) |  |  |  |  |  |  |  |  |
| Cholesterol (g/day) | **1.08** | **0.38** | **1.57** |  |  |  |  |  |
| Salt (g/day) |  |  |  | **0.31** |  |  | **1.73** | **1.23** |
| Alcohol (10 E%) |  |  |  | **0.04** |  |  |  |  |
| Adjusted R^2^ | **0.22** | **0.39** | **0.22** | **0.26** | **ns** | **0.28** | **0.41** | **0.26** |
| *LDL - Low Density Lipoprotein, HDL - High Density Lipoprotein, TC - Total Cholesterol,* | | | | | | | |  |
| *TGC - Triglycerides, LP(a) - Lipoprotein (a), HbA1c - Hemoglobin A1c,* | | | | | |  |  |  |
| *SBP - Systolic Blood Pressure, DBP - Diastolic Blood Pressure, EI - Energy Intake,* | | | | | | |  |  |
| *TEE - Total Energy Expenditure, SFA - Saturated Fatty Acids, BMI - Body Mass Index.* | | | | | | |  |  |

Table S4.

| Summary of linear regression models (weight stable) | | | | | |  |  |  |
| --- | --- | --- | --- | --- | --- | --- | --- | --- |
| Betacoefficients (positive or negative direction of effect) | | | | | |  |  |  |
|  | LDL | HDL | TC | TGC | LP(a) | HbA1c | SBP | DBP |
|  | (mmol/L) | (mmol/L) | (mmol/L) | (mmol/L) | (nmol(L) | (mmol/mol) | (mmHg) | (mmHg) |
| Age (years) |  |  | **0.03** |  |  | **0.11** | **0.70** | **0.26** |
| Male sex |  |  |  | **0.38** |  |  |  |  |
| BMI (kg/m^2^) |  | **-0.05** |  | **0.05** | **5.17** |  |  | **0.68** |
| EI (100 kcal/day) |  |  |  |  |  |  |  |  |
| TEE (100 kcal/day) |  |  |  | **-0.29** |  |  |  |  |
| Carbohydrate (10 E%) | |  |  |  |  |  |  |  |
| Protein (10 E%) |  | **-0.40** |  |  |  |  |  |  |
| Fibre (g/day) | **-0.05** |  |  |  |  |  |  |  |
| SFA (10 E%) |  |  |  |  |  |  |  |  |
| Cholesterol (g/day) | **1.13** | **0.46** | **1.52** |  |  |  |  |  |
| Salt (g/day) |  |  |  |  |  |  | **1.50** | **1.10** |
| Alcohol (10 E%) |  |  |  |  |  |  |  |  |
| Adjusted R^2^ | **0.21** | **0.27** | **0.18** | **0.38** | **0.08** | **0.28** | **0.44** | **0.32** |
| *LDL - Low Density Lipoprotein, HDL - High Density Lipoprotein, TC - Total Cholesterol,* | | | | | | | |  |
| *TGC - Triglycerides, LP(a) - Lipoprotein (a), HbA1c - Hemoglobin A1c,* | | | | | |  |  |  |
| *SBP - Systolic Blood Pressure, DBP - Diastolic Blood Pressure, EI - Energy Intake,* | | | | | | |  |  |
| *TEE - Total Energy Expenditure, SFA - Saturated Fatty Acids, BMI - Body Mass Index.* | | | | | | |  |  |

Figure S1. Distribution of lipids (all)

Legend:

*Distribution of lipids.*

*Box plot shows median and 25 – 75 percentiles.*

*Violin plots shows distribution density*

*LDL – Low Density Lipoprotein, HDL – High Density Lipoprotein*

Figure S2. Distribution of lipids (weight stable)

Legend:

*Distribution of lipids.*

*Box plot shows median and 25 – 75 percentiles.*

*Violin plots shows distribution density*

*LDL – Low Density Lipoprotein, HDL – High Density Lipoprotein*

Figure S3. Distribution of Lp(a) (all)

Legend:

*Distribution of Lp(a).*

*Box plot shows median and 25 – 75 percentiles.*

*Violin plots shows distribution density*

*Lp(a) – Lipoprotein (a)*

Figure S4. Distribution of Lp(a) (weight stable)

Legend:

*Distribution of Lp(a).*

*Box plot shows median and 25 – 75 percentiles.*

*Violin plots shows distribution density*

*Lp(a) – Lipoprotein (a)*

Figure S5. Distribution of Blood pressure (all)

Legend:

*Distribution of systolic and diastolic blood pressure.*

*Box plot shows median and 25 – 75 percentiles.*

*Violin plots shows distribution density*

Figure S6. Distribution of Blood pressure (weight stable)

Legend:

*Distribution of systolic and diastolic blood pressure.*

*Box plot shows median and 25 – 75 percentiles.*

*Violin plots shows distribution density*

Figure S7. Distribution of HbA1c (all)

Legend:

*Distribution of HbA1c.*

*Box plot shows median and 25 – 75 percentiles.*

*Violin plots shows distribution density*

*HbA1c – Hemoglobin A1c*

Figure S8. Distribution of HbA1c (weight stable)

Legend:

*Distribution of HbA1c.*

*Box plot shows median and 25 – 75 percentiles.*

*Violin plots shows distribution density*

*HbA1c – Hemoglobin A1c*
